# 4,5,6,10,11,12,16,17,18,22,23,24-Dodeca­kis­[(meth­oxy­carbon­yl)meth­oxy]-2,8,14,20-tetra­pentyl­resorcin[4]arene

**DOI:** 10.1107/S1600536811047180

**Published:** 2011-11-16

**Authors:** Pramod B. Pansuriya, Holger B. Friedrich, Glenn E. M. Maguire

**Affiliations:** a School of Chemistry, University of KwaZulu-Natal, Durban 4000, South Africa

## Abstract

The title compound, C_84_H_112_O_36_, has a macrocyclic structure. It has 12 (meth­oxy­carbon­yl)meth­oxy ‘head groups’ in the upper rim and exhibits a flattened boat geometry. Intra­molecular C—H⋯O hydrogen bonds occur. In the crystal, inter­molecular C—H⋯O contacts occur. The ‘head groups’ and the pentyl ‘feet’ contain disordered (0.5:0.5 occupancy ratio) atoms.

## Related literature

For applications of resorcin[4]arenes, see: Gerkensmeier *et al.* (1999 ▶); Palmer & Rebek (2005 ▶); Demura *et al.* (2005 ▶); Kulikov *et al.* (2009 ▶); Jin *et al.* (2009 ▶). For structural information, see: McKay *et al.* (2007 ▶); Pansuriya *et al.* (2011 ▶). For the synthesis of tetra­meth­oxy resorcin[4]arenes, see: Gerkensmeier *et al.* (1999 ▶).
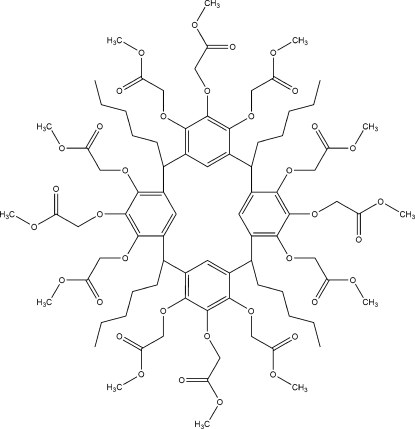


## Experimental

### 

#### Crystal data

C_84_H_112_O_36_
*M* *_r_* = 1697.74
Triclinic, 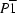

*a* = 13.8526 (7) Å
*b* = 13.9033 (7) Å
*c* = 23.7333 (11) Å
α = 102.231 (1)°
β = 90.865 (1)°
γ = 94.900 (1)°
*V* = 4448.3 (4) Å^3^
*Z* = 2
Mo *K*α radiation
μ = 0.10 mm^−1^
*T* = 173 K
0.49 × 0.32 × 0.28 mm

#### Data collection

Bruker Kappa DUO APEXII diffractometer
Absorption correction: multi-scan (*SADABS*; Bruker, 2006 ▶) *T*_min_ = 0.953, *T*_max_ = 0.973
48747 measured reflections
21987 independent reflections
14127 reflections with *I* > 2σ(*I*)
*R*_int_ = 0.027

#### Refinement

*R*[*F* ^2^ > 2σ(*F* ^2^)] = 0.073
*wR*(*F* ^2^) = 0.251
*S* = 1.05
21987 reflections
1084 parameters
19 restraints
H-atom parameters constrained
Δρ_max_ = 0.93 e Å^−3^
Δρ_min_ = −0.99 e Å^−3^

### 

Data collection: *APEX2* (Bruker, 2006 ▶); cell refinement: *SAINT* (Bruker, 2006 ▶); data reduction: *SAINT*; program(s) used to solve structure: *SHELXS97* (Sheldrick, 2008 ▶); program(s) used to refine structure: *SHELXL97* (Sheldrick, 2008 ▶); molecular graphics: *OLEX2* (Dolomanov *et al.*, 2009 ▶); software used to prepare material for publication: *SHELXL97*.

## Supplementary Material

Crystal structure: contains datablock(s) I, global. DOI: 10.1107/S1600536811047180/hg5135sup1.cif

Structure factors: contains datablock(s) I. DOI: 10.1107/S1600536811047180/hg5135Isup2.hkl

Additional supplementary materials:  crystallographic information; 3D view; checkCIF report

## Figures and Tables

**Table 1:** Hydrogen-bond geometry (Å, °)

*D*—H⋯*A*	*D*—H	H⋯*A*	*D*⋯*A*	*D*—H⋯*A*
C32—H32*B*⋯O18^i^	0.99	2.55	3.304 (4)	133
C34*A*—H34*A*⋯O12^i^	1.16	2.29	3.085 (9)	124
C45—H45*A*⋯O29^ii^	0.98	2.33	3.304 (5)	175
C45—H45*B*⋯O35^iii^	0.98	2.52	3.471 (6)	163
C48*B*—H48*F*⋯O5*A*^iv^	0.98	2.53	3.406 (13)	149
C59*A*—H59*A*⋯O17^v^	0.98	2.37	3.093 (10)	130
C59*A*—H59*D*⋯O17^v^	0.93	2.35	3.093 (10)	137
C62—H62*B*⋯O23^vi^	0.98	2.51	3.415 (6)	153
C73—H73*A*⋯O1^iv^	0.98	2.55	3.491 (4)	160
C79—H79*B*⋯O11^iii^	0.98	2.54	3.443 (5)	153
C84*A*—H84*D*⋯O27^i^	0.98	2.39	3.242 (10)	145

Symmetry codes: (i) 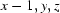; (ii) 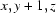; (iii) 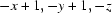; (iv) 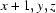; (v) 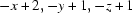; (vi) 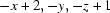.

**Fig. 1.**
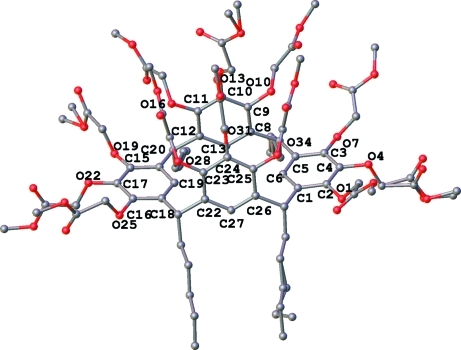
The molecular structure of the title compound with atomic numbering; the hydrogen atoms have been omitted for clarity.

**Fig. 2.**
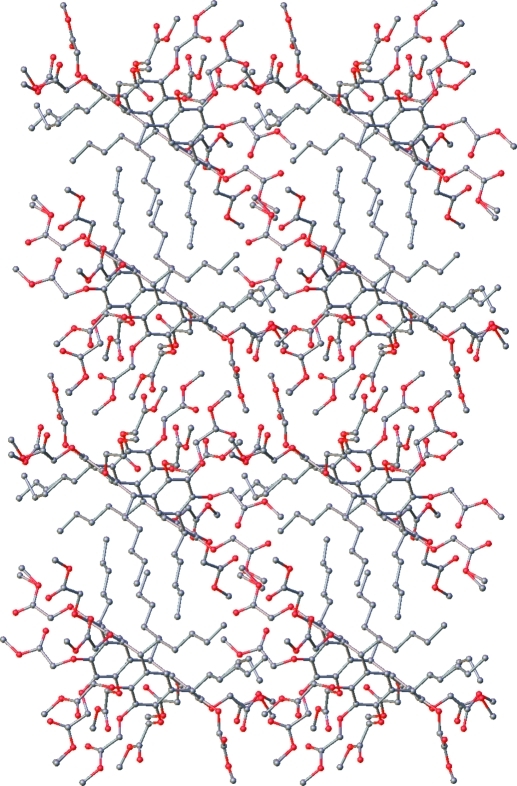
The packing of the title compound in the [010] plane. All hydrogen atoms have been omitted for clarity.

## supplementary crystallographic information

### Comment

In recent years, resorcin[4]arene derivatives with wide ranging features such as constellation isomerism, molecular interaction, synthetic ion channel, and multidented ligand effects (Gerkensmeier *et al.*, 1999; Palmer & Rebek, 2005; Demura *et al.*, 2005; Kulikov *et al.*, 2009; Jin *et al.*, 2009 Have been investigated. Herein, we report the crystal structure of a resorcin[4]arene containing twelve (methoxycarbonyl)methoxy head groups on the upper rim. The title compound is a more flexible analogue compared to resorcin[4]arene structures that we have recently reported (McKay *et al.*, 2007, Pansuriya *et al.*, 2011).

The title compound has an *rccc* (partial boat) configuration (Fig. 1) and is similar to our previously reported tetramethoxy resorcin[4]arene structure which had a boat configuration (Pansuriya *et al.*, 2011). The rings containing C1—C6 and the opposite ring C15—C20, are almost co-planar (dihedral angle 8.36°) whilst the other pair of aromatic rings C8—C13 and C22—C27 are almost parallel (dihedral angle 8.59°). Several non-classical inter and intramolecular hydrogen bonds (C—H···O) are present in the structure.  In the packing structure the (methoxycarbonyl)methoxy groups as well as the feet interlock with each other in the [010] plane (Fig. 2).

### Experimental

In dry degased acetonitrile (100 ml) 2,8,14,20-tetrapentyl-5,11,17,23-tetrahydroxyresorc[4]-arene (Gerkensmeier *et al.*, 1999) (0.499 g, 0.6 mmol) and anhydrous potassium carbonate (2.355 g, 17.1 mmol) were added. The reaction was stirred at 70 °C for ten minutes and then methyl acetyl bromide (1275 µl, 13.8 mmol) was added dropwise. This was allowed to reflux for 24 h and cooled to room temperature. The solvent was reduced in vacuum and the resulting residue extracted with DCM (100 ml). After being washed with 1*M* HCl (50 ml), water (50 ml) and brine (50 ml), the organic layer was dried over anhydrous magnesium sulfate. The solvent was reduced to complete dryness to afford pure product as a white solid (Yield: 0.8 g, 80%).

Crystal suitable for X-ray diffraction analysis were grown at room temperature from a solution of DCM:methanol (2:1).

### Refinement

Several parts of the molecule were disordered and modelled as two positions for each atom with site occupancy factor of 0.50. The disordered moieties are: C48A *versus*. C48B; C59A *versus*. C59B; C82A, C83A and C84A *versus*. C82B, C83B and C84B; C33A, C34A, O5A and O6A *versus*. C33B, C34B, O5B and O6B. The anisotropic displacement parameters of these disordered atoms and the bond distances of the disordered moieties were restrained during refinements. All hydrogen atoms were placed at calculated positions with attach distances ranging from 0.95 Å to 1.00 Å and refined as riding on their parent atoms with *U*_iso_ (H) = 1.2 or 1.5 *U*_eq_ (C).

### Crystal data

**Table:**

C_84_H_112_O_36_	*Z* = 2
*M**_r_* = 1697.74	*F*(000) = 1808
Triclinic, *P*1	*D*_x_ = 1.268 Mg m^−^^3^
Hall symbol: -P 1	Mo *K*α radiation, λ = 0.71073 Å
*a* = 13.8526 (7) Å	Cell parameters from 48747 reflections
*b* = 13.9033 (7) Å	θ = 1.5–28.4°
*c* = 23.7333 (11) Å	µ = 0.10 mm^−^^1^
α = 102.231 (1)°	*T* = 173 K
β = 90.865 (1)°	Block, colourless
γ = 94.900 (1)°	0.49 × 0.32 × 0.28 mm
*V* = 4448.3 (4) Å^3^	

### Data collection

**Table:**

Bruker Kappa DUO APEXII diffractometer	21987 independent reflections
Radiation source: fine-focus sealed tube	14127 reflections with *I* > 2σ(*I*)
graphite	*R*_int_ = 0.027
0.5° φ scans and ω scans	θ_max_ = 28.4°, θ_min_ = 1.5°
Absorption correction: multi-scan (*SADABS*; Bruker, 2006)	*h* = −18→18
*T*_min_ = 0.953, *T*_max_ = 0.973	*k* = −18→18
48747 measured reflections	*l* = −31→31

### Refinement

**Table:**

Refinement on *F*^2^	Primary atom site location: structure-invariant direct methods
Least-squares matrix: full	Secondary atom site location: difference Fourier map
*R*[*F*^2^ > 2σ(*F*^2^)] = 0.073	Hydrogen site location: inferred from neighbouring sites
*wR*(*F*^2^) = 0.251	H-atom parameters constrained
*S* = 1.05	*w* = 1/[σ^2^(*F*_o_^2^) + (0.1324*P*)^2^ + 2.1604*P*] where *P* = (*F*_o_^2^ + 2*F*_c_^2^)/3
21987 reflections	(Δ/σ)_max_ < 0.001
1084 parameters	Δρ_max_ = 0.93 e Å^−^^3^
19 restraints	Δρ_min_ = −0.99 e Å^−^^3^

### Special details

**Table:**

Geometry. All e.s.d.'s (except the e.s.d. in the dihedral angle between two l.s. planes) are estimated using the full covariance matrix. The cell e.s.d.'s are taken into account individually in the estimation of e.s.d.'s in distances, angles and torsion angles; correlations between e.s.d.'s in cell parameters are only used when they are defined by crystal symmetry. An approximate (isotropic) treatment of cell e.s.d.'s is used for estimating e.s.d.'s involving l.s. planes.
Refinement. Refinement of *F*^2^ against ALL reflections. The weighted *R*-factor *wR* and goodness of fit *S* are based on *F*^2^, conventional *R*-factors *R* are based on *F*, with *F* set to zero for negative *F*^2^. The threshold expression of *F*^2^ > σ(*F*^2^) is used only for calculating *R*-factors(gt) *etc*. and is not relevant to the choice of reflections for refinement. *R*-factors based on *F*^2^ are statistically about twice as large as those based on *F*, and *R*- factors based on ALL data will be even larger.

### Fractional atomic coordinates and isotropic or equivalent isotropic displacement parameters (Å^2^)

**Table:**

	*x*	*y*	*z*	*U*_iso_*/*U*_eq_	Occ. (<1)
O1	0.13264 (12)	0.31173 (13)	0.14032 (7)	0.0429 (4)	
O2	0.10243 (17)	0.13560 (17)	0.06370 (10)	0.0651 (6)	
O3	0.0785 (2)	0.2129 (2)	−0.00817 (9)	0.0840 (8)	
O4	0.16273 (13)	0.51494 (14)	0.14819 (7)	0.0464 (4)	
O6B	−0.0588 (11)	0.6228 (14)	0.1755 (8)	0.155 (5)	0.50
O7	0.31777 (14)	0.62305 (13)	0.21628 (7)	0.0467 (4)	
O8	0.4637 (2)	0.7610 (2)	0.20494 (13)	0.0881 (8)	
O9	0.3824 (2)	0.79052 (18)	0.12883 (11)	0.0858 (8)	
O10	0.58545 (13)	0.52770 (12)	0.18926 (7)	0.0424 (4)	
O11	0.6304 (3)	0.5992 (2)	0.09343 (11)	0.1022 (11)	
O12	0.6798 (2)	0.74647 (17)	0.14643 (11)	0.0775 (7)	
O13	0.75594 (12)	0.43942 (15)	0.19019 (7)	0.0467 (4)	
O14	0.89365 (17)	0.3820 (3)	0.11323 (10)	0.0892 (9)	
O15	0.78558 (19)	0.3844 (3)	0.04323 (10)	0.0923 (9)	
O16	0.79734 (11)	0.34439 (14)	0.28147 (7)	0.0451 (4)	
O17	0.92758 (17)	0.47149 (18)	0.35766 (10)	0.0703 (6)	
O18	1.04481 (14)	0.43717 (19)	0.29349 (9)	0.0634 (6)	
O19	0.81969 (12)	0.24638 (13)	0.42267 (7)	0.0437 (4)	
O20	1.06736 (17)	0.3149 (2)	0.40676 (13)	0.0815 (8)	
O21	0.9688 (2)	0.3370 (3)	0.48027 (13)	0.1046 (11)	
O22	0.80037 (13)	0.04236 (14)	0.40250 (8)	0.0475 (4)	
O23	0.92065 (18)	−0.0667 (2)	0.44649 (12)	0.0789 (7)	
O24	0.83115 (18)	−0.05890 (19)	0.52536 (10)	0.0731 (7)	
O25	0.63472 (15)	−0.06190 (13)	0.34663 (9)	0.0549 (5)	
O26	0.72536 (18)	−0.19955 (18)	0.38958 (13)	0.0739 (7)	
O27	0.7890 (2)	−0.25356 (19)	0.30427 (12)	0.0887 (9)	
O28	0.61969 (12)	0.05048 (12)	0.20480 (7)	0.0413 (4)	
O29	0.76894 (14)	0.02241 (16)	0.12940 (9)	0.0601 (5)	
O30	0.84630 (14)	0.16619 (17)	0.17455 (9)	0.0614 (6)	
O31	0.57879 (13)	0.15382 (12)	0.12059 (7)	0.0420 (4)	
O32	0.71822 (16)	0.16864 (18)	0.04556 (9)	0.0675 (6)	
O33	0.65902 (15)	0.02776 (16)	−0.01339 (9)	0.0618 (5)	
O34	0.39248 (13)	0.21417 (13)	0.11147 (7)	0.0449 (4)	
O35	0.48113 (19)	0.22384 (16)	0.00985 (9)	0.0676 (6)	
O36	0.52875 (17)	0.38380 (16)	0.04393 (9)	0.0615 (5)	
C1	0.27237 (16)	0.31799 (17)	0.20164 (10)	0.0360 (5)	
C2	0.21143 (16)	0.36646 (18)	0.17209 (10)	0.0371 (5)	
C3	0.22623 (17)	0.46773 (18)	0.17690 (10)	0.0386 (5)	
C4	0.30425 (18)	0.52186 (17)	0.21001 (10)	0.0377 (5)	
C5	0.36682 (16)	0.47595 (17)	0.24047 (9)	0.0340 (4)	
C6	0.34837 (16)	0.37460 (17)	0.23617 (10)	0.0354 (5)	
H6	0.3890	0.3426	0.2575	0.042*	
C7	0.44980 (17)	0.53857 (16)	0.27734 (9)	0.0346 (5)	
H7	0.4681	0.5944	0.2580	0.042*	
C8	0.54085 (16)	0.48511 (15)	0.27913 (9)	0.0318 (4)	
C9	0.60583 (16)	0.48333 (16)	0.23463 (9)	0.0341 (5)	
C10	0.69133 (16)	0.43676 (17)	0.23376 (9)	0.0356 (5)	
C11	0.71685 (15)	0.39636 (17)	0.28034 (9)	0.0343 (5)	
C12	0.65470 (15)	0.39848 (16)	0.32667 (9)	0.0309 (4)	
C13	0.56720 (15)	0.44136 (15)	0.32418 (9)	0.0308 (4)	
H13	0.5237	0.4406	0.3547	0.037*	
C14	0.68698 (16)	0.35817 (16)	0.37786 (9)	0.0324 (4)	
H14	0.7585	0.3760	0.3828	0.039*	
C15	0.67284 (16)	0.24601 (16)	0.36732 (9)	0.0328 (4)	
C16	0.74106 (17)	0.19455 (18)	0.38985 (10)	0.0372 (5)	
C17	0.72957 (18)	0.09213 (18)	0.38165 (10)	0.0405 (5)	
C18	0.64867 (18)	0.03932 (17)	0.35140 (11)	0.0407 (5)	
C19	0.57916 (17)	0.08802 (17)	0.32812 (9)	0.0350 (5)	
C20	0.59211 (16)	0.19039 (16)	0.33727 (9)	0.0324 (4)	
H20	0.5440	0.2240	0.3225	0.039*	
C21	0.49368 (17)	0.02652 (16)	0.29302 (10)	0.0359 (5)	
H21	0.5174	−0.0379	0.2739	0.043*	
C22	0.46404 (16)	0.07671 (16)	0.24526 (9)	0.0345 (5)	
C23	0.53180 (16)	0.09029 (16)	0.20368 (10)	0.0350 (5)	
C24	0.50914 (17)	0.13776 (16)	0.15997 (9)	0.0362 (5)	
C25	0.41828 (17)	0.17231 (17)	0.15692 (9)	0.0364 (5)	
C26	0.34932 (16)	0.16143 (16)	0.19821 (10)	0.0353 (5)	
C27	0.37446 (16)	0.11397 (16)	0.24166 (10)	0.0341 (4)	
H27	0.3285	0.1067	0.2701	0.041*	
C28	0.25388 (17)	0.20660 (18)	0.19525 (11)	0.0398 (5)	
H28	0.2246	0.1794	0.1557	0.048*	
C29	0.1248 (2)	0.3151 (2)	0.08097 (11)	0.0519 (6)	
H29A	0.1864	0.3446	0.0686	0.062*	
H29B	0.0723	0.3560	0.0745	0.062*	
C30	0.1024 (2)	0.2094 (3)	0.04659 (12)	0.0537 (7)	
C31	0.0546 (4)	0.1173 (4)	−0.04665 (17)	0.1165 (19)	
H31A	0.0384	0.1267	−0.0853	0.175*	
H31B	−0.0010	0.0826	−0.0321	0.175*	
H31C	0.1104	0.0780	−0.0485	0.175*	
C32	0.0816 (2)	0.5415 (3)	0.18191 (14)	0.0644 (8)	
H32A	0.1031	0.5933	0.2163	0.077*	
H32B	0.0523	0.4832	0.1953	0.077*	
C33B	0.0316 (6)	0.6084 (8)	0.1556 (4)	0.060 (2)	0.50
C34B	−0.1142 (10)	0.6568 (10)	0.1266 (7)	0.155 (5)	0.50
H34A	−0.1803	0.6690	0.1383	0.233*	0.50
H34B	−0.1166	0.6050	0.0913	0.233*	0.50
H34C	−0.0805	0.7177	0.1194	0.233*	0.50
C35	0.3204 (4)	0.6603 (3)	0.16515 (16)	0.0885 (14)	
H35A	0.2571	0.6848	0.1583	0.106*	
H35B	0.3317	0.6065	0.1319	0.106*	
C36	0.3975 (3)	0.7411 (2)	0.16959 (14)	0.0651 (8)	
C37	0.4514 (4)	0.8719 (3)	0.12526 (17)	0.0892 (13)	
H37A	0.4317	0.9021	0.0937	0.134*	
H37B	0.5156	0.8481	0.1180	0.134*	
H37C	0.4540	0.9211	0.1617	0.134*	
C38	0.41432 (18)	0.58630 (17)	0.33713 (10)	0.0385 (5)	
H38A	0.3491	0.6083	0.3325	0.046*	
H38B	0.4085	0.5361	0.3612	0.046*	
C39	0.48218 (19)	0.67438 (18)	0.36810 (10)	0.0430 (5)	
H39A	0.5475	0.6523	0.3722	0.052*	
H39B	0.4875	0.7246	0.3441	0.052*	
C40	0.4492 (2)	0.7219 (2)	0.42729 (11)	0.0499 (6)	
H40A	0.4524	0.6745	0.4529	0.060*	
H40B	0.3807	0.7358	0.4240	0.060*	
C41	0.5095 (3)	0.8174 (2)	0.45475 (12)	0.0563 (7)	
H41A	0.5782	0.8038	0.4574	0.068*	
H41B	0.5054	0.8653	0.4295	0.068*	
C42	0.4774 (4)	0.8637 (3)	0.51429 (15)	0.0848 (12)	
H42A	0.5189	0.9246	0.5297	0.127*	
H42B	0.4827	0.8173	0.5398	0.127*	
H42C	0.4100	0.8791	0.5119	0.127*	
C43	0.6397 (3)	0.6229 (2)	0.19571 (13)	0.0613 (8)	
H43A	0.7057	0.6199	0.2115	0.074*	
H43B	0.6075	0.6729	0.2232	0.074*	
C44	0.6463 (3)	0.6519 (2)	0.13949 (14)	0.0647 (8)	
C45	0.6989 (4)	0.7835 (3)	0.0947 (2)	0.0969 (14)	
H45A	0.7234	0.8534	0.1054	0.145*	
H45B	0.6388	0.7766	0.0713	0.145*	
H45C	0.7473	0.7457	0.0724	0.145*	
C46	0.72502 (19)	0.3873 (2)	0.13404 (11)	0.0485 (6)	
H46A	0.6738	0.4209	0.1185	0.058*	
H46B	0.6988	0.3191	0.1349	0.058*	
C47	0.8126 (2)	0.3856 (3)	0.09716 (12)	0.0596 (8)	
C49	0.88925 (19)	0.3759 (3)	0.26271 (12)	0.0618 (8)	
H49A	0.8798	0.4171	0.2342	0.074*	
H49B	0.9209	0.3172	0.2432	0.074*	
C50	0.95370 (19)	0.4331 (2)	0.31051 (12)	0.0469 (6)	
C51	1.1197 (3)	0.4892 (4)	0.33409 (18)	0.0851 (12)	
H51A	1.1824	0.4871	0.3155	0.128*	
H51B	1.1221	0.4578	0.3673	0.128*	
H51C	1.1056	0.5581	0.3471	0.128*	
C52	0.64378 (17)	0.40365 (17)	0.43593 (9)	0.0349 (5)	
H52A	0.5722	0.3920	0.4322	0.042*	
H52B	0.6658	0.3694	0.4657	0.042*	
C53	0.6716 (2)	0.51402 (18)	0.45618 (10)	0.0440 (6)	
H53A	0.7414	0.5280	0.4497	0.053*	
H53B	0.6346	0.5499	0.4325	0.053*	
C54	0.6527 (2)	0.5531 (2)	0.51943 (11)	0.0471 (6)	
H54A	0.5830	0.5385	0.5259	0.056*	
H54B	0.6899	0.5172	0.5430	0.056*	
C55	0.6792 (3)	0.6621 (2)	0.54015 (13)	0.0608 (8)	
H55A	0.6386	0.6981	0.5185	0.073*	
H55B	0.7476	0.6774	0.5311	0.073*	
C56	0.6672 (3)	0.7004 (3)	0.60402 (14)	0.0701 (9)	
H56A	0.6856	0.7717	0.6140	0.105*	
H56B	0.7087	0.6669	0.6260	0.105*	
H56C	0.5993	0.6873	0.6134	0.105*	
C57	0.90769 (18)	0.2393 (2)	0.39250 (12)	0.0471 (6)	
H57A	0.8991	0.2598	0.3554	0.057*	
H57B	0.9234	0.1696	0.3838	0.057*	
C58	0.9896 (2)	0.3023 (2)	0.42670 (15)	0.0582 (7)	
C60	0.7784 (2)	0.0235 (2)	0.45776 (12)	0.0521 (7)	
H60A	0.7130	−0.0117	0.4565	0.063*	
H60B	0.7796	0.0865	0.4866	0.063*	
C61	0.8536 (2)	−0.0389 (2)	0.47394 (14)	0.0549 (7)	
C62	0.8964 (4)	−0.1161 (4)	0.5486 (2)	0.0988 (14)	
H62A	0.8737	−0.1266	0.5859	0.148*	
H62B	0.9613	−0.0808	0.5538	0.148*	
H62C	0.8989	−0.1802	0.5220	0.148*	
C63	0.6997 (3)	−0.1183 (2)	0.31008 (15)	0.0736 (10)	
H63A	0.7548	−0.0743	0.3012	0.088*	
H63B	0.6658	−0.1525	0.2734	0.088*	
C64	0.7369 (2)	−0.1944 (2)	0.34179 (17)	0.0613 (8)	
C65	0.8307 (4)	−0.3291 (3)	0.3290 (2)	0.1020 (15)	
H65A	0.8673	−0.3703	0.2997	0.153*	
H65B	0.7786	−0.3705	0.3421	0.153*	
H65C	0.8742	−0.2972	0.3618	0.153*	
C66	0.41294 (18)	0.00307 (18)	0.33315 (11)	0.0416 (5)	
H66A	0.4433	−0.0118	0.3680	0.050*	
H66B	0.3781	0.0628	0.3457	0.050*	
C67	0.3390 (2)	−0.08324 (19)	0.30649 (13)	0.0494 (6)	
H67A	0.3056	−0.0676	0.2728	0.059*	
H67B	0.3732	−0.1430	0.2927	0.059*	
C68	0.2637 (3)	−0.1046 (2)	0.34966 (17)	0.0691 (9)	
H68A	0.2979	−0.1136	0.3849	0.083*	
H68B	0.2260	−0.0466	0.3608	0.083*	
C69	0.1947 (3)	−0.1945 (3)	0.3272 (3)	0.1151 (14)	
H69A	0.2319	−0.2527	0.3155	0.138*	
H69B	0.1589	−0.1852	0.2926	0.138*	
C71	0.70280 (17)	0.1200 (2)	0.21429 (11)	0.0440 (6)	
H71A	0.7344	0.1199	0.2520	0.053*	
H71B	0.6827	0.1870	0.2156	0.053*	
C72	0.77419 (18)	0.0953 (2)	0.16712 (11)	0.0460 (6)	
C73	0.9223 (2)	0.1517 (3)	0.13299 (15)	0.0713 (10)	
H73A	0.9720	0.2077	0.1421	0.107*	
H73B	0.8947	0.1469	0.0941	0.107*	
H73C	0.9516	0.0906	0.1346	0.107*	
C74	0.57699 (19)	0.07468 (19)	0.07079 (10)	0.0426 (5)	
H74A	0.5147	0.0688	0.0488	0.051*	
H74B	0.5841	0.0114	0.0825	0.051*	
C75	0.6599 (2)	0.0980 (2)	0.03421 (11)	0.0466 (6)	
C76	0.7376 (3)	0.0367 (3)	−0.05216 (16)	0.0840 (12)	
H76A	0.7301	−0.0189	−0.0855	0.126*	
H76B	0.7997	0.0362	−0.0319	0.126*	
H76C	0.7362	0.0989	−0.0652	0.126*	
C77	0.4464 (2)	0.30404 (19)	0.10712 (11)	0.0454 (6)	
H77A	0.5004	0.3191	0.1363	0.054*	
H77B	0.4039	0.3588	0.1151	0.054*	
C78	0.4864 (2)	0.2963 (2)	0.04783 (11)	0.0487 (6)	
C79	0.5725 (3)	0.3904 (3)	−0.01014 (14)	0.0715 (9)	
H79A	0.6012	0.4580	−0.0080	0.107*	
H79B	0.5229	0.3729	−0.0414	0.107*	
H79C	0.6232	0.3447	−0.0177	0.107*	
C80	0.17789 (19)	0.1841 (2)	0.23849 (14)	0.0523 (7)	
H80A	0.2091	0.1992	0.2775	0.063*	
H80B	0.1260	0.2290	0.2389	0.063*	
C48A	0.8589 (6)	0.3402 (9)	0.0006 (3)	0.0923 (9)	0.50
H48A	0.8370	0.3417	−0.0386	0.138*	0.50
H48B	0.8649	0.2716	0.0035	0.138*	0.50
H48C	0.9219	0.3786	0.0095	0.138*	0.50
C59A	1.0468 (7)	0.3747 (9)	0.5269 (4)	0.1046 (11)	0.50
H59A	1.0165	0.3990	0.5636	0.157*	0.50
H59B	1.0882	0.4287	0.5166	0.157*	0.50
H59C	1.0861	0.3209	0.5306	0.157*	0.50
C48B	0.8624 (6)	0.4000 (9)	0.0032 (3)	0.0923 (9)	0.50
H48D	0.8332	0.3995	−0.0347	0.138*	0.50
H48E	0.9056	0.3469	−0.0003	0.138*	0.50
H48F	0.8996	0.4638	0.0180	0.138*	0.50
C59B	1.0539 (7)	0.4097 (9)	0.5074 (4)	0.1046 (11)	0.50
H59D	1.0404	0.4383	0.5476	0.157*	0.50
H59E	1.0633	0.4626	0.4860	0.157*	0.50
H59F	1.1128	0.3751	0.5062	0.157*	0.50
C70	0.1219 (3)	−0.2139 (3)	0.3726 (3)	0.1151 (14)	
H70A	0.0780	−0.2727	0.3563	0.173*	
H70B	0.0842	−0.1568	0.3839	0.173*	
H70C	0.1569	−0.2248	0.4066	0.173*	
C82A	0.0532 (9)	0.0504 (7)	0.2614 (6)	0.0989 (10)	0.50
H82A	0.0030	0.0972	0.2625	0.119*	0.50
H82B	0.0790	0.0575	0.3014	0.119*	0.50
C83B	0.0037 (7)	−0.0223 (7)	0.2817 (5)	0.0989 (10)	0.50
H83A	−0.0426	−0.0128	0.3134	0.119*	0.50
H83B	0.0552	−0.0614	0.2918	0.119*	0.50
C83A	0.0065 (7)	−0.0521 (7)	0.2412 (5)	0.0989 (10)	0.50
H83C	0.0549	−0.1009	0.2398	0.119*	0.50
H83D	−0.0238	−0.0603	0.2023	0.119*	0.50
C84B	−0.0498 (6)	−0.0769 (7)	0.2240 (4)	0.0989 (10)	0.50
H84A	−0.0870	−0.1370	0.2299	0.148*	0.50
H84B	−0.0021	−0.0947	0.1944	0.148*	0.50
H84C	−0.0939	−0.0334	0.2115	0.148*	0.50
C84A	−0.0696 (6)	−0.0663 (7)	0.2845 (5)	0.0989 (10)	0.50
H84D	−0.1002	−0.1342	0.2744	0.148*	0.50
H84E	−0.1188	−0.0199	0.2840	0.148*	0.50
H84F	−0.0390	−0.0541	0.3232	0.148*	0.50
C82B	0.0481 (9)	0.0789 (7)	0.2726 (6)	0.0989 (10)	0.50
H82C	0.0753	0.1175	0.3103	0.119*	0.50
H82D	−0.0050	0.1146	0.2609	0.119*	0.50
C81	0.1314 (3)	0.0780 (3)	0.2262 (2)	0.0989 (10)	
H81A	0.1067	0.0618	0.1856	0.119*	
H81B	0.1836	0.0346	0.2288	0.119*	
O5A	0.0172 (6)	0.5699 (6)	0.0932 (4)	0.106 (3)	0.50
O5B	0.0472 (7)	0.6391 (9)	0.1141 (5)	0.142 (5)	0.50
C33A	0.0037 (8)	0.5812 (9)	0.1459 (6)	0.105 (6)	0.50
O6A	−0.0469 (7)	0.6352 (10)	0.1866 (5)	0.0859 (19)	0.50
C34A	−0.1111 (6)	0.6977 (7)	0.1668 (4)	0.0859 (19)	0.50
H34D	−0.1449	0.7335	0.1996	0.129*	0.50
H34E	−0.1587	0.6571	0.1387	0.129*	0.50
H34F	−0.0737	0.7450	0.1485	0.129*	0.50

### Atomic displacement parameters (Å^2^)

**Table:**

	*U*^11^	*U*^22^	*U*^33^	*U*^12^	*U*^13^	*U*^23^
O1	0.0356 (9)	0.0551 (10)	0.0370 (8)	−0.0006 (7)	−0.0095 (7)	0.0098 (7)
O2	0.0721 (15)	0.0595 (13)	0.0586 (13)	0.0093 (11)	0.0098 (11)	−0.0008 (10)
O3	0.106 (2)	0.0953 (19)	0.0388 (11)	−0.0154 (15)	−0.0057 (12)	−0.0014 (11)
O4	0.0482 (10)	0.0555 (11)	0.0404 (9)	0.0099 (8)	−0.0066 (8)	0.0196 (8)
O6B	0.125 (7)	0.112 (6)	0.231 (11)	0.071 (6)	−0.026 (6)	0.022 (7)
O7	0.0620 (11)	0.0381 (9)	0.0422 (9)	0.0031 (8)	−0.0084 (8)	0.0145 (7)
O8	0.100 (2)	0.0786 (17)	0.0863 (18)	−0.0127 (15)	−0.0174 (16)	0.0284 (15)
O9	0.133 (2)	0.0634 (14)	0.0619 (14)	−0.0249 (15)	−0.0115 (14)	0.0291 (12)
O10	0.0578 (11)	0.0359 (8)	0.0332 (8)	−0.0006 (7)	0.0016 (7)	0.0088 (7)
O11	0.177 (3)	0.0711 (17)	0.0541 (14)	−0.0273 (18)	−0.0237 (17)	0.0209 (13)
O12	0.1058 (19)	0.0533 (13)	0.0777 (16)	−0.0071 (12)	0.0090 (14)	0.0294 (12)
O13	0.0377 (9)	0.0685 (12)	0.0285 (8)	−0.0081 (8)	0.0049 (7)	0.0034 (8)
O14	0.0456 (13)	0.167 (3)	0.0556 (13)	0.0179 (15)	0.0090 (10)	0.0216 (16)
O15	0.0693 (14)	0.170 (3)	0.0415 (10)	0.0178 (16)	0.0131 (9)	0.0290 (15)
O16	0.0279 (8)	0.0666 (12)	0.0386 (9)	0.0077 (7)	0.0033 (7)	0.0047 (8)
O17	0.0631 (14)	0.0711 (14)	0.0623 (13)	−0.0039 (11)	−0.0017 (11)	−0.0137 (11)
O18	0.0353 (10)	0.0942 (16)	0.0617 (13)	−0.0055 (10)	−0.0047 (9)	0.0241 (12)
O19	0.0376 (9)	0.0507 (10)	0.0384 (9)	0.0070 (7)	−0.0098 (7)	−0.0006 (7)
O20	0.0443 (13)	0.0868 (18)	0.119 (2)	−0.0046 (11)	−0.0089 (13)	0.0388 (16)
O21	0.0631 (14)	0.139 (3)	0.0811 (18)	−0.0007 (15)	−0.0243 (13)	−0.0405 (16)
O22	0.0459 (10)	0.0533 (11)	0.0466 (10)	0.0184 (8)	−0.0058 (8)	0.0127 (8)
O23	0.0677 (15)	0.0925 (18)	0.0885 (18)	0.0362 (14)	0.0008 (13)	0.0352 (15)
O24	0.0823 (16)	0.0794 (16)	0.0677 (14)	0.0196 (13)	−0.0118 (12)	0.0344 (13)
O25	0.0630 (12)	0.0341 (9)	0.0673 (12)	0.0115 (8)	−0.0115 (10)	0.0087 (8)
O26	0.0749 (16)	0.0640 (14)	0.0901 (19)	0.0155 (12)	−0.0053 (13)	0.0297 (13)
O27	0.123 (2)	0.0605 (14)	0.0801 (17)	0.0475 (15)	−0.0207 (16)	−0.0041 (12)
O28	0.0348 (8)	0.0406 (9)	0.0451 (9)	0.0050 (7)	0.0010 (7)	0.0008 (7)
O29	0.0497 (11)	0.0590 (12)	0.0600 (12)	0.0065 (9)	0.0083 (9)	−0.0143 (10)
O30	0.0403 (10)	0.0736 (14)	0.0565 (12)	−0.0069 (9)	0.0134 (9)	−0.0134 (10)
O31	0.0464 (9)	0.0415 (9)	0.0328 (8)	−0.0035 (7)	0.0043 (7)	−0.0014 (7)
O32	0.0633 (13)	0.0753 (15)	0.0506 (12)	−0.0166 (11)	0.0139 (10)	−0.0084 (10)
O33	0.0573 (12)	0.0663 (13)	0.0495 (11)	0.0030 (10)	0.0080 (9)	−0.0146 (9)
O34	0.0531 (10)	0.0437 (9)	0.0367 (9)	−0.0041 (8)	−0.0109 (8)	0.0099 (7)
O35	0.1036 (18)	0.0573 (13)	0.0397 (10)	0.0062 (12)	0.0008 (11)	0.0056 (9)
O36	0.0785 (15)	0.0582 (12)	0.0456 (11)	−0.0073 (10)	0.0035 (10)	0.0117 (9)
C1	0.0340 (11)	0.0359 (11)	0.0359 (11)	0.0014 (9)	−0.0039 (9)	0.0039 (9)
C2	0.0330 (11)	0.0443 (12)	0.0332 (11)	0.0034 (9)	−0.0024 (9)	0.0070 (9)
C3	0.0390 (12)	0.0478 (13)	0.0312 (11)	0.0079 (10)	−0.0016 (9)	0.0120 (10)
C4	0.0448 (13)	0.0373 (12)	0.0316 (11)	0.0044 (10)	0.0027 (9)	0.0089 (9)
C5	0.0361 (11)	0.0355 (11)	0.0291 (10)	0.0026 (9)	−0.0006 (8)	0.0041 (8)
C6	0.0335 (11)	0.0370 (11)	0.0346 (11)	0.0041 (9)	−0.0049 (9)	0.0052 (9)
C7	0.0388 (12)	0.0314 (10)	0.0318 (10)	0.0008 (9)	−0.0019 (9)	0.0037 (8)
C8	0.0330 (11)	0.0282 (10)	0.0306 (10)	−0.0022 (8)	−0.0026 (8)	0.0004 (8)
C9	0.0390 (12)	0.0325 (11)	0.0277 (10)	−0.0043 (9)	−0.0016 (8)	0.0031 (8)
C10	0.0327 (11)	0.0401 (12)	0.0297 (10)	−0.0060 (9)	0.0038 (8)	0.0011 (9)
C11	0.0270 (10)	0.0401 (12)	0.0311 (10)	−0.0013 (8)	−0.0010 (8)	−0.0012 (9)
C12	0.0287 (10)	0.0329 (10)	0.0277 (10)	−0.0013 (8)	−0.0017 (8)	0.0003 (8)
C13	0.0290 (10)	0.0319 (10)	0.0282 (10)	−0.0015 (8)	0.0012 (8)	0.0009 (8)
C14	0.0297 (10)	0.0354 (11)	0.0294 (10)	0.0019 (8)	−0.0011 (8)	0.0011 (8)
C15	0.0338 (11)	0.0353 (11)	0.0272 (10)	0.0079 (8)	0.0003 (8)	0.0004 (8)
C16	0.0348 (11)	0.0442 (12)	0.0308 (10)	0.0074 (9)	−0.0044 (9)	0.0028 (9)
C17	0.0435 (13)	0.0435 (13)	0.0357 (11)	0.0154 (10)	−0.0031 (10)	0.0068 (10)
C18	0.0462 (13)	0.0337 (11)	0.0411 (12)	0.0090 (10)	−0.0031 (10)	0.0040 (9)
C19	0.0371 (11)	0.0357 (11)	0.0308 (10)	0.0046 (9)	−0.0035 (9)	0.0037 (9)
C20	0.0317 (10)	0.0354 (11)	0.0289 (10)	0.0069 (8)	−0.0020 (8)	0.0031 (8)
C21	0.0389 (12)	0.0307 (11)	0.0367 (11)	0.0030 (9)	−0.0011 (9)	0.0044 (9)
C22	0.0368 (11)	0.0304 (10)	0.0324 (10)	−0.0004 (8)	−0.0037 (9)	−0.0003 (8)
C23	0.0332 (11)	0.0318 (11)	0.0351 (11)	0.0013 (8)	−0.0032 (9)	−0.0029 (8)
C24	0.0405 (12)	0.0330 (11)	0.0307 (10)	−0.0035 (9)	0.0007 (9)	−0.0002 (8)
C25	0.0408 (12)	0.0341 (11)	0.0313 (10)	−0.0027 (9)	−0.0067 (9)	0.0029 (9)
C26	0.0353 (11)	0.0306 (10)	0.0363 (11)	−0.0017 (8)	−0.0068 (9)	0.0013 (9)
C27	0.0333 (11)	0.0320 (11)	0.0344 (11)	−0.0010 (8)	−0.0024 (9)	0.0028 (8)
C28	0.0344 (12)	0.0387 (12)	0.0434 (12)	−0.0013 (9)	−0.0095 (10)	0.0051 (10)
C29	0.0517 (15)	0.0635 (17)	0.0391 (13)	0.0016 (13)	−0.0092 (11)	0.0101 (12)
C30	0.0435 (15)	0.072 (2)	0.0391 (13)	0.0032 (13)	0.0040 (11)	−0.0015 (13)
C31	0.151 (5)	0.121 (4)	0.052 (2)	−0.033 (3)	−0.005 (2)	−0.022 (2)
C32	0.0660 (19)	0.076 (2)	0.0591 (18)	0.0308 (16)	0.0038 (15)	0.0217 (16)
C33B	0.031 (4)	0.085 (7)	0.075 (5)	0.012 (4)	0.007 (4)	0.040 (5)
C34B	0.125 (7)	0.112 (6)	0.231 (11)	0.071 (6)	−0.026 (6)	0.022 (7)
C35	0.128 (3)	0.076 (2)	0.066 (2)	−0.036 (2)	−0.030 (2)	0.0446 (19)
C36	0.098 (3)	0.0474 (16)	0.0461 (16)	0.0030 (16)	−0.0016 (17)	0.0039 (13)
C37	0.139 (4)	0.0513 (19)	0.071 (2)	−0.021 (2)	0.012 (2)	0.0100 (17)
C38	0.0410 (12)	0.0374 (12)	0.0347 (11)	0.0073 (9)	−0.0010 (9)	0.0012 (9)
C39	0.0493 (14)	0.0378 (12)	0.0377 (12)	0.0049 (10)	−0.0013 (10)	−0.0013 (10)
C40	0.0567 (16)	0.0461 (14)	0.0414 (13)	0.0085 (12)	0.0046 (12)	−0.0048 (11)
C41	0.078 (2)	0.0455 (15)	0.0406 (14)	0.0053 (14)	0.0006 (13)	−0.0015 (11)
C42	0.121 (3)	0.064 (2)	0.0566 (19)	0.010 (2)	0.017 (2)	−0.0172 (16)
C43	0.087 (2)	0.0431 (15)	0.0505 (16)	−0.0134 (14)	0.0028 (15)	0.0111 (12)
C44	0.087 (2)	0.0494 (16)	0.0578 (18)	−0.0105 (15)	−0.0114 (16)	0.0181 (14)
C45	0.126 (4)	0.078 (3)	0.098 (3)	−0.011 (2)	−0.003 (3)	0.052 (2)
C46	0.0421 (14)	0.0633 (17)	0.0336 (12)	−0.0011 (12)	0.0051 (10)	−0.0019 (11)
C47	0.0452 (16)	0.094 (2)	0.0373 (13)	0.0063 (15)	0.0070 (12)	0.0085 (14)
C49	0.0312 (13)	0.105 (3)	0.0439 (15)	0.0020 (14)	0.0041 (11)	0.0059 (15)
C50	0.0412 (13)	0.0519 (15)	0.0478 (14)	0.0013 (11)	−0.0024 (11)	0.0124 (12)
C51	0.054 (2)	0.113 (3)	0.086 (3)	−0.030 (2)	−0.0198 (18)	0.035 (2)
C52	0.0380 (12)	0.0375 (11)	0.0262 (10)	0.0037 (9)	−0.0014 (8)	0.0002 (8)
C53	0.0537 (15)	0.0384 (12)	0.0347 (12)	0.0003 (11)	0.0001 (10)	−0.0025 (9)
C54	0.0508 (15)	0.0464 (14)	0.0371 (12)	0.0049 (11)	0.0022 (11)	−0.0068 (10)
C55	0.076 (2)	0.0436 (15)	0.0527 (16)	0.0025 (14)	0.0028 (15)	−0.0108 (12)
C56	0.073 (2)	0.0630 (19)	0.0570 (18)	0.0024 (16)	0.0021 (16)	−0.0254 (15)
C57	0.0383 (13)	0.0549 (15)	0.0479 (14)	0.0084 (11)	−0.0042 (11)	0.0094 (12)
C58	0.0447 (16)	0.0565 (17)	0.072 (2)	0.0068 (13)	−0.0126 (14)	0.0106 (15)
C60	0.0568 (16)	0.0580 (16)	0.0442 (14)	0.0186 (13)	−0.0061 (12)	0.0122 (12)
C61	0.0550 (17)	0.0511 (16)	0.0612 (17)	0.0082 (13)	−0.0139 (14)	0.0177 (13)
C62	0.111 (3)	0.098 (3)	0.108 (3)	0.024 (3)	−0.015 (3)	0.065 (3)
C63	0.108 (3)	0.0476 (17)	0.0611 (19)	0.0343 (18)	−0.0197 (19)	−0.0059 (14)
C64	0.0600 (18)	0.0381 (14)	0.079 (2)	0.0095 (12)	−0.0238 (16)	−0.0015 (14)
C65	0.117 (4)	0.071 (3)	0.122 (4)	0.047 (3)	−0.019 (3)	0.017 (2)
C66	0.0452 (13)	0.0385 (12)	0.0407 (12)	0.0013 (10)	0.0003 (10)	0.0083 (10)
C67	0.0494 (15)	0.0386 (13)	0.0584 (16)	−0.0021 (11)	−0.0012 (12)	0.0090 (12)
C68	0.0627 (19)	0.0501 (17)	0.093 (3)	0.0002 (14)	0.0207 (18)	0.0137 (16)
C69	0.0762 (19)	0.0654 (17)	0.197 (4)	−0.0128 (14)	0.046 (2)	0.017 (2)
C71	0.0348 (12)	0.0506 (14)	0.0408 (13)	0.0022 (10)	−0.0006 (10)	−0.0027 (10)
C72	0.0365 (13)	0.0538 (15)	0.0438 (13)	0.0066 (11)	−0.0002 (10)	0.0005 (11)
C73	0.0426 (16)	0.091 (2)	0.067 (2)	−0.0053 (15)	0.0179 (14)	−0.0103 (17)
C74	0.0451 (13)	0.0406 (12)	0.0365 (12)	0.0009 (10)	−0.0017 (10)	−0.0032 (10)
C75	0.0485 (14)	0.0508 (15)	0.0368 (12)	0.0083 (12)	−0.0003 (11)	−0.0007 (11)
C76	0.077 (2)	0.099 (3)	0.061 (2)	0.005 (2)	0.0257 (18)	−0.0157 (19)
C77	0.0550 (15)	0.0402 (13)	0.0393 (12)	0.0015 (11)	−0.0015 (11)	0.0062 (10)
C78	0.0585 (16)	0.0483 (15)	0.0396 (13)	0.0061 (12)	−0.0070 (12)	0.0100 (11)
C79	0.082 (2)	0.086 (2)	0.0485 (17)	−0.0080 (19)	0.0041 (16)	0.0244 (16)
C80	0.0321 (12)	0.0546 (16)	0.0726 (19)	−0.0001 (11)	−0.0024 (12)	0.0212 (14)
C48A	0.0693 (14)	0.170 (3)	0.0415 (10)	0.0178 (16)	0.0131 (9)	0.0290 (15)
C59A	0.0631 (14)	0.139 (3)	0.0811 (18)	−0.0007 (15)	−0.0243 (13)	−0.0405 (16)
C48B	0.0693 (14)	0.170 (3)	0.0415 (10)	0.0178 (16)	0.0131 (9)	0.0290 (15)
C59B	0.0631 (14)	0.139 (3)	0.0811 (18)	−0.0007 (15)	−0.0243 (13)	−0.0405 (16)
C70	0.0762 (19)	0.0654 (17)	0.197 (4)	−0.0128 (14)	0.046 (2)	0.017 (2)
C82A	0.0645 (14)	0.092 (2)	0.143 (3)	−0.0269 (14)	−0.0132 (17)	0.045 (2)
C83B	0.0645 (14)	0.092 (2)	0.143 (3)	−0.0269 (14)	−0.0132 (17)	0.045 (2)
C83A	0.0645 (14)	0.092 (2)	0.143 (3)	−0.0269 (14)	−0.0132 (17)	0.045 (2)
C84B	0.0645 (14)	0.092 (2)	0.143 (3)	−0.0269 (14)	−0.0132 (17)	0.045 (2)
C84A	0.0645 (14)	0.092 (2)	0.143 (3)	−0.0269 (14)	−0.0132 (17)	0.045 (2)
C82B	0.0645 (14)	0.092 (2)	0.143 (3)	−0.0269 (14)	−0.0132 (17)	0.045 (2)
C81	0.0645 (14)	0.092 (2)	0.143 (3)	−0.0269 (14)	−0.0132 (17)	0.045 (2)
O5A	0.100 (5)	0.121 (6)	0.117 (6)	0.031 (5)	−0.037 (5)	0.066 (5)
O5B	0.134 (8)	0.219 (11)	0.140 (8)	0.123 (8)	0.081 (6)	0.140 (8)
C33A	0.082 (9)	0.060 (6)	0.174 (13)	−0.002 (5)	−0.075 (9)	0.036 (7)
O6A	0.065 (3)	0.102 (4)	0.109 (4)	0.049 (3)	0.026 (3)	0.045 (4)
C34A	0.065 (3)	0.102 (4)	0.109 (4)	0.049 (3)	0.026 (3)	0.045 (4)

### Geometric parameters (Å, °)

**Table:**

O1—C2	1.389 (3)	C40—C41	1.521 (4)
O1—C29	1.422 (3)	C40—H40A	0.9900
O2—C30	1.181 (4)	C40—H40B	0.9900
O3—C30	1.348 (4)	C41—C42	1.513 (4)
O3—C31	1.453 (5)	C41—H41A	0.9900
O4—C3	1.392 (3)	C41—H41B	0.9900
O4—C32	1.419 (4)	C42—H42A	0.9800
O6B—C33B	1.361 (14)	C42—H42B	0.9800
O6B—C34B	1.558 (15)	C42—H42C	0.9800
O7—C4	1.379 (3)	C43—C44	1.476 (4)
O7—C35	1.417 (3)	C43—H43A	0.9900
O8—C36	1.207 (4)	C43—H43B	0.9900
O9—C36	1.324 (4)	C45—H45A	0.9800
O9—C37	1.435 (4)	C45—H45B	0.9800
O10—C9	1.386 (3)	C45—H45C	0.9800
O10—C43	1.442 (3)	C46—C47	1.506 (4)
O11—C44	1.186 (4)	C46—H46A	0.9900
O12—C44	1.331 (4)	C46—H46B	0.9900
O12—C45	1.448 (4)	C49—C50	1.477 (4)
O13—C10	1.382 (3)	C49—H49A	0.9900
O13—C46	1.416 (3)	C49—H49B	0.9900
O14—C47	1.188 (4)	C51—H51A	0.9800
O15—C47	1.325 (4)	C51—H51B	0.9800
O15—C48B	1.469 (8)	C51—H51C	0.9800
O15—C48A	1.519 (9)	C52—C53	1.522 (3)
O16—C11	1.382 (3)	C52—H52A	0.9900
O16—C49	1.420 (3)	C52—H52B	0.9900
O17—C50	1.208 (3)	C53—C54	1.519 (3)
O18—C50	1.331 (3)	C53—H53A	0.9900
O18—C51	1.440 (4)	C53—H53B	0.9900
O19—C16	1.384 (3)	C54—C55	1.503 (4)
O19—C57	1.423 (3)	C54—H54A	0.9900
O20—C58	1.197 (4)	C54—H54B	0.9900
O21—C58	1.306 (4)	C55—C56	1.513 (4)
O21—C59A	1.512 (9)	C55—H55A	0.9900
O21—C59B	1.525 (10)	C55—H55B	0.9900
O22—C17	1.390 (3)	C56—H56A	0.9800
O22—C60	1.423 (3)	C56—H56B	0.9800
O23—C61	1.187 (4)	C56—H56C	0.9800
O24—C61	1.343 (4)	C57—C58	1.489 (4)
O24—C62	1.431 (4)	C57—H57A	0.9900
O25—C18	1.384 (3)	C57—H57B	0.9900
O25—C63	1.426 (4)	C60—C61	1.506 (4)
O26—C64	1.164 (4)	C60—H60A	0.9900
O27—C64	1.342 (4)	C60—H60B	0.9900
O27—C65	1.462 (4)	C62—H62A	0.9800
O28—C23	1.382 (3)	C62—H62B	0.9800
O28—C71	1.421 (3)	C62—H62C	0.9800
O29—C72	1.198 (3)	C63—C64	1.540 (4)
O30—C72	1.324 (3)	C63—H63A	0.9900
O30—C73	1.451 (3)	C63—H63B	0.9900
O31—C24	1.390 (3)	C65—H65A	0.9800
O31—C74	1.432 (3)	C65—H65B	0.9800
O32—C75	1.199 (3)	C65—H65C	0.9800
O33—C75	1.326 (3)	C66—C67	1.529 (3)
O33—C76	1.450 (4)	C66—H66A	0.9900
O34—C25	1.385 (3)	C66—H66B	0.9900
O34—C77	1.424 (3)	C67—C68	1.527 (4)
O35—C78	1.197 (3)	C67—H67A	0.9900
O36—C78	1.327 (3)	C67—H67B	0.9900
O36—C79	1.445 (4)	C68—C69	1.503 (5)
C1—C2	1.392 (3)	C68—H68A	0.9900
C1—C6	1.400 (3)	C68—H68B	0.9900
C1—C28	1.523 (3)	C69—C70	1.537 (6)
C2—C3	1.385 (3)	C69—H69A	0.9900
C3—C4	1.392 (3)	C69—H69B	0.9900
C4—C5	1.397 (3)	C71—C72	1.509 (4)
C5—C6	1.392 (3)	C71—H71A	0.9900
C5—C7	1.524 (3)	C71—H71B	0.9900
C6—H6	0.9500	C73—H73A	0.9800
C7—C8	1.522 (3)	C73—H73B	0.9800
C7—C38	1.540 (3)	C73—H73C	0.9800
C7—H7	1.0000	C74—C75	1.504 (4)
C8—C9	1.396 (3)	C74—H74A	0.9900
C8—C13	1.396 (3)	C74—H74B	0.9900
C9—C10	1.396 (3)	C76—H76A	0.9800
C10—C11	1.396 (3)	C76—H76B	0.9800
C11—C12	1.404 (3)	C76—H76C	0.9800
C12—C13	1.402 (3)	C77—C78	1.506 (4)
C12—C14	1.518 (3)	C77—H77A	0.9900
C13—H13	0.9500	C77—H77B	0.9900
C14—C15	1.521 (3)	C79—H79A	0.9800
C14—C52	1.542 (3)	C79—H79B	0.9800
C14—H14	1.0000	C79—H79C	0.9800
C15—C20	1.397 (3)	C80—C81	1.525 (5)
C15—C16	1.398 (3)	C80—H80A	0.9900
C16—C17	1.391 (3)	C80—H80B	0.9900
C17—C18	1.386 (4)	C48A—H48A	0.9800
C18—C19	1.395 (3)	C48A—H48B	0.9800
C19—C20	1.389 (3)	C48A—H48C	0.9800
C19—C21	1.524 (3)	C59A—H59A	0.9800
C20—H20	0.9500	C59A—H59B	0.9800
C21—C22	1.520 (3)	C59A—H59C	0.9800
C21—C66	1.538 (3)	C48B—H48D	0.9800
C21—H21	1.0000	C48B—H48E	0.9800
C22—C27	1.393 (3)	C48B—H48F	0.9800
C22—C23	1.403 (3)	C59B—H59D	0.9800
C23—C24	1.388 (3)	C59B—H59E	0.9800
C24—C25	1.391 (3)	C59B—H59F	0.9800
C25—C26	1.401 (3)	C70—H70A	0.9800
C26—C27	1.392 (3)	C70—H70B	0.9800
C26—C28	1.518 (3)	C70—H70C	0.9800
C27—H27	0.9500	C82A—C81	1.453 (11)
C28—C80	1.540 (4)	C82A—C83A	1.491 (11)
C28—H28	1.0000	C82A—H82A	0.9900
C29—C30	1.526 (4)	C82A—H82B	0.9900
C29—H29A	0.9900	C83B—C82B	1.545 (11)
C29—H29B	0.9900	C83B—C84B	1.560 (12)
C31—H31A	0.9800	C83B—H83A	0.9900
C31—H31B	0.9800	C83B—H83B	0.9900
C31—H31C	0.9800	C83A—C84A	1.512 (11)
C32—C33B	1.442 (11)	C83A—H83C	0.9900
C32—C33A	1.571 (13)	C83A—H83D	0.9900
C32—H32A	0.9900	C84B—H84A	0.9800
C32—H32B	0.9900	C84B—H84B	0.9800
C33B—O5B	1.169 (10)	C84B—H84C	0.9800
C34B—H34A	0.9800	C84A—H84D	0.9800
C34B—H34B	0.9800	C84A—H84E	0.9800
C34B—H34C	0.9800	C84A—H84F	0.9800
C35—C36	1.468 (5)	C82B—C81	1.606 (12)
C35—H35A	0.9900	C82B—H82C	0.9900
C35—H35B	0.9900	C82B—H82D	0.9900
C37—H37A	0.9800	C81—H81A	0.9900
C37—H37B	0.9800	C81—H81B	0.9900
C37—H37C	0.9800	O5A—C33A	1.246 (14)
C38—C39	1.525 (3)	C33A—O6A	1.338 (14)
C38—H38A	0.9900	O6A—C34A	1.435 (10)
C38—H38B	0.9900	C34A—H34D	0.9800
C39—C40	1.515 (3)	C34A—H34E	0.9800
C39—H39A	0.9900	C34A—H34F	0.9800
C39—H39B	0.9900		
			
C2—O1—C29	117.03 (19)	O18—C51—H51A	109.5
C30—O3—C31	115.0 (3)	O18—C51—H51B	109.5
C3—O4—C32	111.32 (19)	H51A—C51—H51B	109.5
C33B—O6B—C34B	105.2 (12)	O18—C51—H51C	109.5
C4—O7—C35	117.1 (2)	H51A—C51—H51C	109.5
C36—O9—C37	117.7 (3)	H51B—C51—H51C	109.5
C9—O10—C43	111.29 (19)	C53—C52—C14	113.71 (19)
C44—O12—C45	117.1 (3)	C53—C52—H52A	108.8
C10—O13—C46	116.97 (18)	C14—C52—H52A	108.8
C47—O15—C48B	117.3 (4)	C53—C52—H52B	108.8
C47—O15—C48A	111.8 (4)	C14—C52—H52B	108.8
C48B—O15—C48A	31.7 (5)	H52A—C52—H52B	107.7
C11—O16—C49	122.9 (2)	C54—C53—C52	113.5 (2)
C50—O18—C51	118.5 (3)	C54—C53—H53A	108.9
C16—O19—C57	112.58 (18)	C52—C53—H53A	108.9
C58—O21—C59A	122.0 (5)	C54—C53—H53B	108.9
C58—O21—C59B	107.1 (5)	C52—C53—H53B	108.9
C59A—O21—C59B	28.2 (6)	H53A—C53—H53B	107.7
C17—O22—C60	111.9 (2)	C55—C54—C53	114.2 (2)
C61—O24—C62	115.8 (3)	C55—C54—H54A	108.7
C18—O25—C63	115.3 (2)	C53—C54—H54A	108.7
C64—O27—C65	113.1 (3)	C55—C54—H54B	108.7
C23—O28—C71	115.44 (18)	C53—C54—H54B	108.7
C72—O30—C73	115.7 (2)	H54A—C54—H54B	107.6
C24—O31—C74	113.09 (18)	C54—C55—C56	114.6 (3)
C75—O33—C76	116.3 (3)	C54—C55—H55A	108.6
C25—O34—C77	116.58 (18)	C56—C55—H55A	108.6
C78—O36—C79	116.8 (2)	C54—C55—H55B	108.6
C2—C1—C6	118.0 (2)	C56—C55—H55B	108.6
C2—C1—C28	119.1 (2)	H55A—C55—H55B	107.6
C6—C1—C28	122.9 (2)	C55—C56—H56A	109.5
C3—C2—O1	120.9 (2)	C55—C56—H56B	109.5
C3—C2—C1	120.6 (2)	H56A—C56—H56B	109.5
O1—C2—C1	118.4 (2)	C55—C56—H56C	109.5
C2—C3—C4	120.2 (2)	H56A—C56—H56C	109.5
C2—C3—O4	119.6 (2)	H56B—C56—H56C	109.5
C4—C3—O4	120.2 (2)	O19—C57—C58	111.6 (2)
O7—C4—C3	120.5 (2)	O19—C57—H57A	109.3
O7—C4—C5	118.6 (2)	C58—C57—H57A	109.3
C3—C4—C5	120.8 (2)	O19—C57—H57B	109.3
C6—C5—C4	117.6 (2)	C58—C57—H57B	109.3
C6—C5—C7	123.4 (2)	H57A—C57—H57B	108.0
C4—C5—C7	119.0 (2)	O20—C58—O21	124.7 (3)
C5—C6—C1	122.6 (2)	O20—C58—C57	122.1 (3)
C5—C6—H6	118.7	O21—C58—C57	113.2 (3)
C1—C6—H6	118.7	O22—C60—C61	107.9 (2)
C8—C7—C5	113.69 (18)	O22—C60—H60A	110.1
C8—C7—C38	114.30 (18)	C61—C60—H60A	110.1
C5—C7—C38	110.53 (19)	O22—C60—H60B	110.1
C8—C7—H7	105.8	C61—C60—H60B	110.1
C5—C7—H7	105.8	H60A—C60—H60B	108.4
C38—C7—H7	105.8	O23—C61—O24	125.4 (3)
C9—C8—C13	116.8 (2)	O23—C61—C60	126.9 (3)
C9—C8—C7	118.77 (19)	O24—C61—C60	107.8 (3)
C13—C8—C7	124.38 (19)	O24—C62—H62A	109.5
O10—C9—C8	119.8 (2)	O24—C62—H62B	109.5
O10—C9—C10	118.2 (2)	H62A—C62—H62B	109.5
C8—C9—C10	121.9 (2)	O24—C62—H62C	109.5
O13—C10—C11	119.4 (2)	H62A—C62—H62C	109.5
O13—C10—C9	120.5 (2)	H62B—C62—H62C	109.5
C11—C10—C9	119.7 (2)	O25—C63—C64	108.1 (3)
O16—C11—C10	123.28 (19)	O25—C63—H63A	110.1
O16—C11—C12	116.2 (2)	C64—C63—H63A	110.1
C10—C11—C12	120.2 (2)	O25—C63—H63B	110.1
C13—C12—C11	118.01 (19)	C64—C63—H63B	110.1
C13—C12—C14	123.25 (19)	H63A—C63—H63B	108.4
C11—C12—C14	118.70 (19)	O26—C64—O27	125.4 (3)
C8—C13—C12	123.25 (19)	O26—C64—C63	127.6 (3)
C8—C13—H13	118.4	O27—C64—C63	107.0 (3)
C12—C13—H13	118.4	O27—C65—H65A	109.5
C12—C14—C15	113.19 (17)	O27—C65—H65B	109.5
C12—C14—C52	115.35 (18)	H65A—C65—H65B	109.5
C15—C14—C52	109.74 (18)	O27—C65—H65C	109.5
C12—C14—H14	105.9	H65A—C65—H65C	109.5
C15—C14—H14	105.9	H65B—C65—H65C	109.5
C52—C14—H14	105.9	C67—C66—C21	115.0 (2)
C20—C15—C16	117.2 (2)	C67—C66—H66A	108.5
C20—C15—C14	123.04 (19)	C21—C66—H66A	108.5
C16—C15—C14	119.74 (19)	C67—C66—H66B	108.5
O19—C16—C17	119.2 (2)	C21—C66—H66B	108.5
O19—C16—C15	119.6 (2)	H66A—C66—H66B	107.5
C17—C16—C15	121.2 (2)	C68—C67—C66	111.9 (2)
C18—C17—O22	119.9 (2)	C68—C67—H67A	109.2
C18—C17—C16	120.1 (2)	C66—C67—H67A	109.2
O22—C17—C16	120.0 (2)	C68—C67—H67B	109.2
O25—C18—C17	119.6 (2)	C66—C67—H67B	109.2
O25—C18—C19	119.8 (2)	H67A—C67—H67B	107.9
C17—C18—C19	120.5 (2)	C69—C68—C67	113.9 (3)
C20—C19—C18	118.3 (2)	C69—C68—H68A	108.8
C20—C19—C21	123.12 (19)	C67—C68—H68A	108.8
C18—C19—C21	118.6 (2)	C69—C68—H68B	108.8
C19—C20—C15	122.8 (2)	C67—C68—H68B	108.8
C19—C20—H20	118.6	H68A—C68—H68B	107.7
C15—C20—H20	118.6	C68—C69—C70	111.9 (4)
C22—C21—C19	109.33 (18)	C68—C69—H69A	109.2
C22—C21—C66	116.00 (19)	C70—C69—H69A	109.2
C19—C21—C66	110.29 (19)	C68—C69—H69B	109.2
C22—C21—H21	106.9	C70—C69—H69B	109.2
C19—C21—H21	106.9	H69A—C69—H69B	107.9
C66—C21—H21	106.9	O28—C71—C72	111.0 (2)
C27—C22—C23	117.7 (2)	O28—C71—H71A	109.4
C27—C22—C21	123.7 (2)	C72—C71—H71A	109.4
C23—C22—C21	118.5 (2)	O28—C71—H71B	109.4
O28—C23—C24	120.2 (2)	C72—C71—H71B	109.4
O28—C23—C22	118.9 (2)	H71A—C71—H71B	108.0
C24—C23—C22	120.8 (2)	O29—C72—O30	124.5 (2)
C23—C24—O31	119.7 (2)	O29—C72—C71	126.2 (2)
C23—C24—C25	120.0 (2)	O30—C72—C71	109.3 (2)
O31—C24—C25	120.2 (2)	O30—C73—H73A	109.5
O34—C25—C24	120.6 (2)	O30—C73—H73B	109.5
O34—C25—C26	118.5 (2)	H73A—C73—H73B	109.5
C24—C25—C26	120.8 (2)	O30—C73—H73C	109.5
C27—C26—C25	117.8 (2)	H73A—C73—H73C	109.5
C27—C26—C28	123.5 (2)	H73B—C73—H73C	109.5
C25—C26—C28	118.6 (2)	O31—C74—C75	107.5 (2)
C26—C27—C22	122.9 (2)	O31—C74—H74A	110.2
C26—C27—H27	118.6	C75—C74—H74A	110.2
C22—C27—H27	118.6	O31—C74—H74B	110.2
C26—C28—C1	110.00 (18)	C75—C74—H74B	110.2
C26—C28—C80	115.9 (2)	H74A—C74—H74B	108.5
C1—C28—C80	109.4 (2)	O32—C75—O33	124.5 (3)
C26—C28—H28	107.0	O32—C75—C74	126.1 (2)
C1—C28—H28	107.0	O33—C75—C74	109.4 (2)
C80—C28—H28	107.0	O33—C76—H76A	109.5
O1—C29—C30	107.8 (2)	O33—C76—H76B	109.5
O1—C29—H29A	110.1	H76A—C76—H76B	109.5
C30—C29—H29A	110.1	O33—C76—H76C	109.5
O1—C29—H29B	110.1	H76A—C76—H76C	109.5
C30—C29—H29B	110.1	H76B—C76—H76C	109.5
H29A—C29—H29B	108.5	O34—C77—C78	110.7 (2)
O2—C30—O3	124.0 (3)	O34—C77—H77A	109.5
O2—C30—C29	127.6 (3)	C78—C77—H77A	109.5
O3—C30—C29	108.3 (3)	O34—C77—H77B	109.5
O3—C31—H31A	109.5	C78—C77—H77B	109.5
O3—C31—H31B	109.5	H77A—C77—H77B	108.1
H31A—C31—H31B	109.5	O35—C78—O36	124.8 (3)
O3—C31—H31C	109.5	O35—C78—C77	126.3 (3)
H31A—C31—H31C	109.5	O36—C78—C77	108.9 (2)
H31B—C31—H31C	109.5	O36—C79—H79A	109.5
O4—C32—C33B	107.7 (4)	O36—C79—H79B	109.5
O4—C32—C33A	110.8 (6)	H79A—C79—H79B	109.5
C33B—C32—C33A	19.7 (6)	O36—C79—H79C	109.5
O4—C32—H32A	109.5	H79A—C79—H79C	109.5
C33B—C32—H32A	93.4	H79B—C79—H79C	109.5
C33A—C32—H32A	109.5	C81—C80—C28	115.1 (3)
O4—C32—H32B	109.5	C81—C80—H80A	108.5
C33B—C32—H32B	126.9	C28—C80—H80A	108.5
C33A—C32—H32B	109.5	C81—C80—H80B	108.5
H32A—C32—H32B	108.1	C28—C80—H80B	108.5
O5B—C33B—O6B	113.3 (11)	H80A—C80—H80B	107.5
O5B—C33B—C32	131.3 (8)	O15—C48A—H48A	109.5
O6B—C33B—C32	113.9 (9)	O15—C48A—H48B	109.5
O6B—C34B—H34A	109.5	H48A—C48A—H48B	109.5
O6B—C34B—H34B	109.5	O15—C48A—H48C	109.5
H34A—C34B—H34B	109.5	H48A—C48A—H48C	109.5
O6B—C34B—H34C	109.5	H48B—C48A—H48C	109.5
H34A—C34B—H34C	109.5	O21—C59A—H59A	109.5
H34B—C34B—H34C	109.5	O21—C59A—H59B	109.5
O7—C35—C36	110.8 (3)	H59A—C59A—H59B	109.5
O7—C35—H35A	109.5	O21—C59A—H59C	109.5
C36—C35—H35A	109.5	H59A—C59A—H59C	109.5
O7—C35—H35B	109.5	H59B—C59A—H59C	109.5
C36—C35—H35B	109.5	O15—C48B—H48D	109.5
H35A—C35—H35B	108.1	O15—C48B—H48E	109.5
O8—C36—O9	124.8 (3)	H48D—C48B—H48E	109.5
O8—C36—C35	126.1 (3)	O15—C48B—H48F	109.5
O9—C36—C35	109.1 (3)	H48D—C48B—H48F	109.5
O9—C37—H37A	109.5	H48E—C48B—H48F	109.5
O9—C37—H37B	109.5	O21—C59B—H59D	109.5
H37A—C37—H37B	109.5	O21—C59B—H59E	109.5
O9—C37—H37C	109.5	H59D—C59B—H59E	109.5
H37A—C37—H37C	109.5	O21—C59B—H59F	109.5
H37B—C37—H37C	109.5	H59D—C59B—H59F	109.5
C39—C38—C7	112.7 (2)	H59E—C59B—H59F	109.5
C39—C38—H38A	109.1	C69—C70—H70A	109.5
C7—C38—H38A	109.1	C69—C70—H70B	109.5
C39—C38—H38B	109.1	H70A—C70—H70B	109.5
C7—C38—H38B	109.1	C69—C70—H70C	109.5
H38A—C38—H38B	107.8	H70A—C70—H70C	109.5
C40—C39—C38	113.6 (2)	H70B—C70—H70C	109.5
C40—C39—H39A	108.8	C81—C82A—C83A	114.8 (9)
C38—C39—H39A	108.8	C81—C82A—H82A	108.6
C40—C39—H39B	108.8	C83A—C82A—H82A	108.6
C38—C39—H39B	108.8	C81—C82A—H82B	108.6
H39A—C39—H39B	107.7	C83A—C82A—H82B	108.6
C39—C40—C41	113.4 (2)	H82A—C82A—H82B	107.5
C39—C40—H40A	108.9	C82B—C83B—C84B	107.3 (9)
C41—C40—H40A	108.9	C82B—C83B—H83A	110.2
C39—C40—H40B	108.9	C84B—C83B—H83A	110.2
C41—C40—H40B	108.9	C82B—C83B—H83B	110.2
H40A—C40—H40B	107.7	C84B—C83B—H83B	110.2
C42—C41—C40	113.2 (3)	H83A—C83B—H83B	108.5
C42—C41—H41A	108.9	C82A—C83A—C84A	106.1 (9)
C40—C41—H41A	108.9	C82A—C83A—H83C	110.5
C42—C41—H41B	108.9	C84A—C83A—H83C	110.5
C40—C41—H41B	108.9	C82A—C83A—H83D	110.5
H41A—C41—H41B	107.7	C84A—C83A—H83D	110.5
C41—C42—H42A	109.5	H83C—C83A—H83D	108.7
C41—C42—H42B	109.5	C83B—C84B—H84A	109.5
H42A—C42—H42B	109.5	C83B—C84B—H84B	109.5
C41—C42—H42C	109.5	H84A—C84B—H84B	109.5
H42A—C42—H42C	109.5	C83B—C84B—H84C	109.5
H42B—C42—H42C	109.5	H84A—C84B—H84C	109.5
O10—C43—C44	110.3 (2)	H84B—C84B—H84C	109.5
O10—C43—H43A	109.6	C83A—C84A—H84D	109.5
C44—C43—H43A	109.6	C83A—C84A—H84E	109.5
O10—C43—H43B	109.6	H84D—C84A—H84E	109.5
C44—C43—H43B	109.6	C83A—C84A—H84F	109.5
H43A—C43—H43B	108.1	H84D—C84A—H84F	109.5
O11—C44—O12	122.7 (3)	H84E—C84A—H84F	109.5
O11—C44—C43	126.4 (3)	C83B—C82B—C81	117.2 (8)
O12—C44—C43	110.7 (3)	C83B—C82B—H82C	108.0
O12—C45—H45A	109.5	C81—C82B—H82C	108.0
O12—C45—H45B	109.5	C83B—C82B—H82D	108.0
H45A—C45—H45B	109.5	C81—C82B—H82D	108.0
O12—C45—H45C	109.5	H82C—C82B—H82D	107.2
H45A—C45—H45C	109.5	C82A—C81—C80	119.8 (6)
H45B—C45—H45C	109.5	C82A—C81—C82B	15.5 (6)
O13—C46—C47	106.8 (2)	C80—C81—C82B	104.4 (5)
O13—C46—H46A	110.4	C82A—C81—H81A	107.4
C47—C46—H46A	110.4	C80—C81—H81A	107.4
O13—C46—H46B	110.4	C82B—C81—H81A	114.0
C47—C46—H46B	110.4	C82A—C81—H81B	107.4
H46A—C46—H46B	108.6	C80—C81—H81B	107.4
O14—C47—O15	124.7 (3)	C82B—C81—H81B	116.3
O14—C47—C46	125.4 (3)	H81A—C81—H81B	106.9
O15—C47—C46	109.8 (3)	O5A—C33A—O6A	139.2 (12)
O16—C49—C50	112.8 (2)	O5A—C33A—C32	116.0 (10)
O16—C49—H49A	109.0	O6A—C33A—C32	103.2 (10)
C50—C49—H49A	109.0	C33A—O6A—C34A	115.9 (10)
O16—C49—H49B	109.0	O6A—C34A—H34D	109.5
C50—C49—H49B	109.0	O6A—C34A—H34E	109.5
H49A—C49—H49B	107.8	H34D—C34A—H34E	109.5
O17—C50—O18	125.1 (3)	O6A—C34A—H34F	109.5
O17—C50—C49	125.2 (3)	H34D—C34A—H34F	109.5
O18—C50—C49	109.7 (2)	H34E—C34A—H34F	109.5
			
C29—O1—C2—C3	58.8 (3)	O31—C24—C25—O34	6.5 (3)
C29—O1—C2—C1	−124.6 (2)	C23—C24—C25—C26	1.2 (3)
C6—C1—C2—C3	0.1 (3)	O31—C24—C25—C26	−176.5 (2)
C28—C1—C2—C3	−180.0 (2)	O34—C25—C26—C27	176.28 (19)
C6—C1—C2—O1	−176.4 (2)	C24—C25—C26—C27	−0.8 (3)
C28—C1—C2—O1	3.5 (3)	O34—C25—C26—C28	−7.0 (3)
O1—C2—C3—C4	178.5 (2)	C24—C25—C26—C28	175.9 (2)
C1—C2—C3—C4	2.0 (4)	C25—C26—C27—C22	−0.6 (3)
O1—C2—C3—O4	−1.8 (3)	C28—C26—C27—C22	−177.1 (2)
C1—C2—C3—O4	−178.2 (2)	C23—C22—C27—C26	1.5 (3)
C32—O4—C3—C2	86.8 (3)	C21—C22—C27—C26	178.7 (2)
C32—O4—C3—C4	−93.5 (3)	C27—C26—C28—C1	116.1 (2)
C35—O7—C4—C3	−53.3 (4)	C25—C26—C28—C1	−60.4 (3)
C35—O7—C4—C5	130.7 (3)	C27—C26—C28—C80	−8.7 (3)
C2—C3—C4—O7	−178.2 (2)	C25—C26—C28—C80	174.8 (2)
O4—C3—C4—O7	2.1 (3)	C2—C1—C28—C26	146.5 (2)
C2—C3—C4—C5	−2.2 (3)	C6—C1—C28—C26	−33.6 (3)
O4—C3—C4—C5	178.0 (2)	C2—C1—C28—C80	−85.1 (3)
O7—C4—C5—C6	176.3 (2)	C6—C1—C28—C80	94.9 (3)
C3—C4—C5—C6	0.3 (3)	C2—O1—C29—C30	133.5 (2)
O7—C4—C5—C7	−2.5 (3)	C31—O3—C30—O2	−1.8 (5)
C3—C4—C5—C7	−178.5 (2)	C31—O3—C30—C29	−179.8 (3)
C4—C5—C6—C1	1.9 (3)	O1—C29—C30—O2	−8.3 (4)
C7—C5—C6—C1	−179.3 (2)	O1—C29—C30—O3	169.6 (2)
C2—C1—C6—C5	−2.1 (3)	C3—O4—C32—C33B	167.3 (5)
C28—C1—C6—C5	177.9 (2)	C3—O4—C32—C33A	−172.1 (5)
C6—C5—C7—C8	35.2 (3)	C34B—O6B—C33B—O5B	11.2 (19)
C4—C5—C7—C8	−146.0 (2)	C34B—O6B—C33B—C32	−156.6 (10)
C6—C5—C7—C38	−94.9 (3)	O4—C32—C33B—O5B	1.1 (17)
C4—C5—C7—C38	83.9 (2)	C33A—C32—C33B—O5B	−101 (3)
C5—C7—C8—C9	83.9 (2)	O4—C32—C33B—O6B	166.1 (11)
C38—C7—C8—C9	−147.9 (2)	C33A—C32—C33B—O6B	64 (2)
C5—C7—C8—C13	−99.6 (2)	C4—O7—C35—C36	−138.0 (3)
C38—C7—C8—C13	28.6 (3)	C37—O9—C36—O8	2.0 (6)
C43—O10—C9—C8	101.5 (3)	C37—O9—C36—C35	−178.9 (4)
C43—O10—C9—C10	−79.2 (3)	O7—C35—C36—O8	15.1 (6)
C13—C8—C9—O10	−178.33 (18)	O7—C35—C36—O9	−163.9 (3)
C7—C8—C9—O10	−1.6 (3)	C8—C7—C38—C39	69.2 (3)
C13—C8—C9—C10	2.5 (3)	C5—C7—C38—C39	−161.0 (2)
C7—C8—C9—C10	179.23 (19)	C7—C38—C39—C40	−179.4 (2)
C46—O13—C10—C11	119.3 (3)	C38—C39—C40—C41	−172.4 (2)
C46—O13—C10—C9	−67.6 (3)	C39—C40—C41—C42	−179.0 (3)
O10—C9—C10—O13	3.2 (3)	C9—O10—C43—C44	159.1 (3)
C8—C9—C10—O13	−177.6 (2)	C45—O12—C44—O11	−1.4 (6)
O10—C9—C10—C11	176.31 (19)	C45—O12—C44—C43	174.4 (4)
C8—C9—C10—C11	−4.5 (3)	O10—C43—C44—O11	−16.1 (6)
C49—O16—C11—C10	43.8 (3)	O10—C43—C44—O12	168.3 (3)
C49—O16—C11—C12	−142.5 (2)	C10—O13—C46—C47	−169.9 (2)
O13—C10—C11—O16	−10.5 (3)	C48B—O15—C47—O14	−12.9 (8)
C9—C10—C11—O16	176.3 (2)	C48A—O15—C47—O14	21.6 (7)
O13—C10—C11—C12	176.11 (19)	C48B—O15—C47—C46	169.7 (6)
C9—C10—C11—C12	2.9 (3)	C48A—O15—C47—C46	−155.8 (5)
O16—C11—C12—C13	−173.41 (19)	O13—C46—C47—O14	33.3 (5)
C10—C11—C12—C13	0.5 (3)	O13—C46—C47—O15	−149.3 (3)
O16—C11—C12—C14	9.0 (3)	C11—O16—C49—C50	95.5 (3)
C10—C11—C12—C14	−177.2 (2)	C51—O18—C50—O17	1.5 (5)
C9—C8—C13—C12	1.1 (3)	C51—O18—C50—C49	−179.5 (3)
C7—C8—C13—C12	−175.48 (19)	O16—C49—C50—O17	−17.7 (5)
C11—C12—C13—C8	−2.5 (3)	O16—C49—C50—O18	163.2 (3)
C14—C12—C13—C8	175.00 (19)	C12—C14—C52—C53	−62.3 (3)
C13—C12—C14—C15	102.7 (2)	C15—C14—C52—C53	168.46 (19)
C11—C12—C14—C15	−79.8 (2)	C14—C52—C53—C54	−164.5 (2)
C13—C12—C14—C52	−24.8 (3)	C52—C53—C54—C55	−179.7 (2)
C11—C12—C14—C52	152.7 (2)	C53—C54—C55—C56	−176.0 (3)
C12—C14—C15—C20	−38.9 (3)	C16—O19—C57—C58	175.2 (2)
C52—C14—C15—C20	91.6 (2)	C59A—O21—C58—O20	−16.0 (8)
C12—C14—C15—C16	143.3 (2)	C59B—O21—C58—O20	10.5 (7)
C52—C14—C15—C16	−86.3 (2)	C59A—O21—C58—C57	160.5 (6)
C57—O19—C16—C17	76.0 (3)	C59B—O21—C58—C57	−173.0 (6)
C57—O19—C16—C15	−106.3 (2)	O19—C57—C58—O20	−172.1 (3)
C20—C15—C16—O19	−176.48 (19)	O19—C57—C58—O21	11.3 (4)
C14—C15—C16—O19	1.5 (3)	C17—O22—C60—C61	174.5 (2)
C20—C15—C16—C17	1.2 (3)	C62—O24—C61—O23	2.0 (5)
C14—C15—C16—C17	179.1 (2)	C62—O24—C61—C60	−178.6 (3)
C60—O22—C17—C18	−86.5 (3)	O22—C60—C61—O23	1.2 (5)
C60—O22—C17—C16	95.1 (3)	O22—C60—C61—O24	−178.3 (2)
O19—C16—C17—C18	176.7 (2)	C18—O25—C63—C64	133.8 (3)
C15—C16—C17—C18	−1.0 (4)	C65—O27—C64—O26	1.0 (5)
O19—C16—C17—O22	−4.9 (3)	C65—O27—C64—C63	178.9 (3)
C15—C16—C17—O22	177.4 (2)	O25—C63—C64—O26	−8.9 (5)
C63—O25—C18—C17	−71.0 (3)	O25—C63—C64—O27	173.3 (3)
C63—O25—C18—C19	112.0 (3)	C22—C21—C66—C67	73.6 (3)
O22—C17—C18—O25	5.8 (4)	C19—C21—C66—C67	−161.4 (2)
C16—C17—C18—O25	−175.9 (2)	C21—C66—C67—C68	177.6 (2)
O22—C17—C18—C19	−177.2 (2)	C66—C67—C68—C69	−174.8 (3)
C16—C17—C18—C19	1.1 (4)	C67—C68—C69—C70	178.9 (4)
O25—C18—C19—C20	175.5 (2)	C23—O28—C71—C72	−125.3 (2)
C17—C18—C19—C20	−1.5 (4)	C73—O30—C72—O29	0.3 (4)
O25—C18—C19—C21	−5.6 (3)	C73—O30—C72—C71	178.6 (3)
C17—C18—C19—C21	177.4 (2)	O28—C71—C72—O29	−7.2 (4)
C18—C19—C20—C15	1.8 (3)	O28—C71—C72—O30	174.4 (2)
C21—C19—C20—C15	−177.1 (2)	C24—O31—C74—C75	−175.9 (2)
C16—C15—C20—C19	−1.6 (3)	C76—O33—C75—O32	3.1 (5)
C14—C15—C20—C19	−179.5 (2)	C76—O33—C75—C74	−177.0 (3)
C20—C19—C21—C22	32.4 (3)	O31—C74—C75—O32	1.8 (4)
C18—C19—C21—C22	−146.5 (2)	O31—C74—C75—O33	−178.1 (2)
C20—C19—C21—C66	−96.3 (3)	C25—O34—C77—C78	126.8 (2)
C18—C19—C21—C66	84.8 (3)	C79—O36—C78—O35	−0.9 (4)
C19—C21—C22—C27	−115.9 (2)	C79—O36—C78—C77	179.4 (3)
C66—C21—C22—C27	9.5 (3)	O34—C77—C78—O35	−5.0 (4)
C19—C21—C22—C23	61.3 (3)	O34—C77—C78—O36	174.6 (2)
C66—C21—C22—C23	−173.3 (2)	C26—C28—C80—C81	−71.2 (3)
C71—O28—C23—C24	65.9 (3)	C1—C28—C80—C81	163.7 (3)
C71—O28—C23—C22	−117.9 (2)	C81—C82A—C83A—C84A	178.2 (10)
C27—C22—C23—O28	−177.33 (18)	C84B—C83B—C82B—C81	−64.7 (12)
C21—C22—C23—O28	5.3 (3)	C83A—C82A—C81—C80	173.5 (8)
C27—C22—C23—C24	−1.1 (3)	C83A—C82A—C81—C82B	169 (5)
C21—C22—C23—C24	−178.5 (2)	C28—C80—C81—C82A	−175.0 (8)
O28—C23—C24—O31	−6.4 (3)	C28—C80—C81—C82B	−173.7 (6)
C22—C23—C24—O31	177.47 (19)	C83B—C82B—C81—C82A	10 (3)
O28—C23—C24—C25	175.99 (19)	C83B—C82B—C81—C80	−165.5 (9)
C22—C23—C24—C25	−0.2 (3)	O4—C32—C33A—O5A	10.1 (12)
C74—O31—C24—C23	90.4 (2)	C33B—C32—C33A—O5A	95 (2)
C74—O31—C24—C25	−92.0 (3)	O4—C32—C33A—O6A	−157.9 (8)
C77—O34—C25—C24	−64.7 (3)	C33B—C32—C33A—O6A	−73.3 (19)
C77—O34—C25—C26	118.2 (2)	O5A—C33A—O6A—C34A	4(2)
C23—C24—C25—O34	−175.9 (2)	C32—C33A—O6A—C34A	167.7 (9)

### Hydrogen-bond geometry (Å, °)

**Table:**

*D*—H···*A*	*D*—H	H···*A*	*D*···*A*	*D*—H···*A*
C32—H32B···O18^i^	0.99	2.55	3.304 (4)	133
C34A—H34A···O12^i^	1.16	2.29	3.085 (9)	124
C45—H45A···O29^ii^	0.98	2.33	3.304 (5)	175
C45—H45B···O35^iii^	0.98	2.52	3.471 (6)	163
C48B—H48F···O5A^iv^	0.98	2.53	3.406 (13)	149
C59A—H59A···O17^v^	0.98	2.37	3.093 (10)	130
C59A—H59D···O17^v^	0.93	2.35	3.093 (10)	137
C62—H62B···O23^vi^	0.98	2.51	3.415 (6)	153
C73—H73A···O1^iv^	0.98	2.55	3.491 (4)	160
C79—H79B···O11^iii^	0.98	2.54	3.443 (5)	153
C84A—H84D···O27^i^	0.98	2.39	3.242 (10)	145

Symmetry codes: (i) *x*−1, *y*, *z*; (ii) *x*, *y*+1, *z*; (iii) −*x*+1, −*y*+1, −*z*; (iv) *x*+1, *y*, *z*; (v) −*x*+2, −*y*+1, −*z*+1; (vi) −*x*+2, −*y*, −*z*+1.
